# Optical Methods for Determining the Phagocytic Activity Profile of CD206-Positive Macrophages Extracted from Bronchoalveolar Lavage by Specific Mannosylated Polymeric Ligands

**DOI:** 10.3390/polym17010065

**Published:** 2024-12-30

**Authors:** Igor D. Zlotnikov, Alexander A. Ezhov, Natalia I. Kolganova, Dmitry Yurievich Ovsyannikov, Natalya G. Belogurova, Elena V. Kudryashova

**Affiliations:** 1 Faculty of Chemistry, Lomonosov Moscow State University, Leninskie Gory, 1/3, 119991 Moscow, Russia; zlotnikovid@my.msu.ru (I.D.Z.);; 2 Faculty of Physics, Lomonosov Moscow State University, Leninskie Gory, 1/2, 119991 Moscow, Russia; 3 Institute of Medicine, RUDN University, 6 Miklukho-Maklaya St, 117198 Moscow, Russia

**Keywords:** mannosylated polymeric ligands, macrophage, CD206 receptor, bronchoalveolar lavage, phenotype

## Abstract

Macrophage (Mph) polarization and functional activity play an important role in the development of inflammatory lung conditions. The previously widely used bimodal classification of Mph into M1 and M2 does not adequately reflect the full range of changes in polarization and functional diversity observed in Mph in response to various stimuli and disease states. Here, we have developed a model for the direct assessment of Mph from bronchial alveolar lavage fluid (BALF) functional alterations, in terms of phagocytosis activity, depending on external stimuli, such as exposure to a range of bacteria (*E. coli*, *B. subtilis* and *L. fermentum*). We have employed polymeric mannosylated ligands (the “trapping ligand”) specifically targeting the CD206 receptor to selectively isolate activated Mph from the BALF of patients with pulmonary inflammatory conditions: primary ciliary dyskinesia (PCD), pneumonia and bronchial asthma. An “imaging ligand” allows for the subsequent visualization of the isolated cells using a sandwich technique. Five model strains of *E. coli*, MH-1, JM109, BL21, W3110 and ATCC25922, as well as *B. subtilis* and *L. fermentum* strains, each exhibiting distinct properties and expressing red fluorescent protein (RFP), were used as a phagocytosis substrate. Fluorometric, FTIR- and confocal laser scanning microscopy (CLSM) assessments of the phagocytic response of Mph to these bacterial cells were performed. Mph absorbed different strains of *E. coli* with different activities due to the difference in the surface villosity of bacterial cells (pili and fimbriae, as well as signal patterns). In the presence of other competitor cells (like those of *Lactobacilli*), the phagocytic activity of Mph is changed between two and five times and strongly dependent on the bacterial strain. The relative phagocytic activity indexes obtained for BALF-Mph in comparison with that obtained for model human CD206+ Mph in the M1 polarization state (derived from THP-1 monocyte cultures) were considered as a set of parameters to define the Mph polarization profile from the BALF of patients. Mannan as a marker determining the selectivity of the binding to the CD 206 mannose receptor of Mph significantly inhibited the phagocytosis of *E. coli* and *B. subtilis* in cases of pneumonia, suggesting an important role of CD206 overexpression in acute inflammation. Conversely, *L. fermentum* binding was enhanced in PCD, possibly reflecting altered macrophage responsiveness in chronic lung diseases. Our approach based on the profiling of Mph from patient BALF samples in terms of phagocytosis for a range of model bacterial strains is important for the subsequent detailed study of the factors determining dangerous conditions and resistance to existing therapeutic options.

## 1. Introduction

Macrophages constitute an important component in the immune response to a wide range of conditions, encompassing autoimmune, infectious, inflammation and fibrotic diseases, among others [1,2,3,4,5]. The key mechanism responsible for maintaining tissue integrity and initiating tissue response to injury is the complex activity of tissue macrophages, which remove dying cells and bacteria, as well as produce a wide range of biologically active substances essential for tissue function. Recent data indicate a pronounced heterogeneity of tissue macrophages, both in terms of their origin in the tissue and in relation to cytokines, growth factors and enzymes that remodel the extracellular matrix [6,7]. In barrier tissues, including those of the lungs, macrophages can be categorized into several functional types. Classically activated macrophages (M1) protect the body against microorganisms by triggering an inflammatory response through the secretion of pro-inflammatory cytokines [8,9,10,11,12,13,14]. Alternatively activated (M2) macrophages regulate the resolution after injury by producing anti-inflammatory cytokines. M2 macrophages are subdivided into subpopulations of functionally and phenotypically related cells: M2a, M2b and M2c [15,16,17,18,19,20]. M2a cells are considered as alternatively activated; they are characterized by the increased expression of the CD206-mannose receptor. It has been shown that M2a macrophages have anti-inflammatory properties and are involved in tissue remodeling. M2b macrophages are involved in the regulation of the immune response and the involvement of T-regulatory cells. M2c macrophages express CD163 and have pronounced anti-inflammatory activity. In cases of chronic or persistent inflammation, macrophages may be exposed to constant activation signals. It is suggested that an increased expression of the M2 macrophage phenotype is associated to fibrosis tissue remodeling in the heart and lungs. The balance between different types of macrophages is essential for maintaining cellular tissue homeostasis. When this balance is disrupted, pathological fibrosis or chronic inflammation can occur.

Recent data suggest that the heterogeneity of macrophages, in terms of their surface receptor repertoire and range of excreted factors, goes far beyond the classical M1/M2 paradigm [11,12,15,19,21,22,23]. Macrophage phenotype varies significantly among individuals and is strongly influenced by various factors such as overall immune status, type of infection and degree of inflammation [1]. Bronchoalveolar lavage fluid (BALF) analysis is a widely used medical diagnostic tool [24,25,26]. There are many examples of studies confirming the clinical significance of macrophage analysis from BALF. According to flow cytometry data, the proportion of CD14++CD16 macrophages from BALF in the subgroup of patients with pulmonary sarcoidosis was lower than in that in healthy people, and the proportion of CD14++CD16+ in the subgroup was higher [27]. A nomogram model has been devised that incorporates the analysis of neutrophils derived from BALF and procalcitonin levels, which has been demonstrated to be a highly valuable tool in predicting the risk of pulmonary infection. This model holds substantial promise in assisting clinicians in making well-informed treatment decisions [28]. In certain pathological conditions, such as alveolar proteinosis, diffuse alveolar hemorrhage (DAH) or pulmonary infections, BALF can provide strong support for diagnosis. Moreover, in other scenarios, the analysis of BALF fluid can guide clinicians towards a specific diagnosis [29].

The bronchoalveolar lavage fluid contains not only epithelial cells, but also cells that have migrated from the circulatory system, such as eosinophils, basophils, lymphocytes and neutrophils [24,25,30,31]. The specific cellular composition of BALF is indicative of various pathological conditions affecting the lungs. Thus, elucidating the state of macrophage polarization, their phenotypic characteristics and phagocytic activity represents a critical endeavor in the realm of advanced diagnostic procedures for bacterial respiratory infections and other macrophage-related pathologies. The elucidation of the mechanisms of a favorable scenario of tissue response to inflammation, which involves complete recovery, requires the development of new criteria and new research methods.

Here, we isolated the activated CD206+ macrophages from BALF samples of patients to analyze the phagocytic activity of macrophages in relation to a number of model bacterial cells. In the future, this approach could be useful for searching for a correlation with the clinical manifestations. We used the recently developed mannosylated polymeric ligands specifically targeting the CD206 receptors of macrophages [32,33]. We suggested applying these ligands containing specific oligo-(trimannoside) and poly-(mannoside) residues as a tool for assessing the state of CD206+ macrophages and further, based on the developed analysis method of macrophages’ phagocytic activity, determining the distinguishing features for a number of inflammation conditions, such as bacterial infections, bronchial asthma, etc. Recently, we employed the concept of macrophage typing based on a set of ligands, allowing us to elucidate the intricate relationship between disease, macrophage phenotype and ligand-binding activity [34].

In this study, we used these specific mannoside ligands to specifically isolate activated macrophages from BALF samples of the patients with a chronic inflammatory process caused by primary ciliary dyskinesia (PCD), pneumonia and bronchial asthma. PCD is a rare hereditary disease from the group of ciliopathies, based on a defect in the ultrastructure of the cilia of the respiratory tract epithelium, leading to the damage of their motoric function with the development of chronic inflammation [35]. Pneumonia is acute infectious inflammation of the lung tissue, mainly of bacterial origin. The disease manifests with severe symptoms, such as coughing, chest pain, a very high body temperature, etc. [36,37]. Bronchial asthma is a chronic inflammatory disease of the airways, characterized by recurrent episodes of breathlessness, wheezing, chest tightness and coughing, especially at night or in the early morning. It is usually triggered by various allergens, infections, stress, physical exertion or other factors that cause inflammation and the narrowing of the airways [38,39,40].

We assess BALF macrophage phagocytosis in response to various model strains of *E. coli*, *B. subtilis* and *L. fermentum* characterized by distinct origins and surface antigens as one of the parameters for macrophage functional status typing. Presumably, this parameter will characterize the degree and nature of inflammation and, along with other parameters, will be used for inflammatory diseases of the respiratory tract

Here, we develop a new method for evaluating the phagocytic activity of CD206 macrophages in the BALF of patients with acute or chronic inflammation states (PCD, pneumonia or bronchial asthma). We believe that the proposed approach based on a comparative index of phagocytic activity in relation to a set of model bacterial cells opens possibilities for assessing the status of inflammation and determining dangerous conditions in the case of lung inflammatory diseases, including autoimmune, infectious, bronchial asthma and oncological diseases.

## 2. Materials and Methods

### 2.1. Reagents

Mannan (46 kDa), polyethyleneimine 1.8 kDa (PEI1.8), 2-hydroxypropyl-β-cyclodextrin (HPCD), FITC, putrescine (put), NaBH_3_CN and activated PEG 5 kDa (N-succinimidyl ester of mono-methoxy poly (ethylene glycol)) were purchased from Sigma Aldrich (St. Louis, MO, USA). Mannotriose-di-(N-acetyl-D-glucosamine) (triMan) was obtained from Dayang Chem (Hangzhou, China) Co., Ltd. Carbonyldiimidazole (CDI) was obtained from GL Biochem Ltd. (Shanghai, China). Other chemicals (salts and acids) were from Reakhim Production (Moscow, Russia).

### 2.2. Synthesis and Characterization of Ligands for Macrophage CD206 Receptors

We propose employing polyethylene glycol (PEG) modified with trimannoside residues as a macrophage “trapping ligand”. This polymer may be applied to the surface of biosensors to isolate macrophages from biological samples. The trapping ligand also serves as an infrared (IR) marker, exhibiting a distinct and analytically meaningful spectral band at 1090 cm^−1^. This band differs from those characteristics of major functional groups in biopolymers such as hydroxyl (-OH), carboxyl (-COOH), amino, aromatic, amide and phosphate biopolymers. This feature makes it suitable for IR surface mapping applications.

An “imaging ligand” for macrophage visualization offers a viable alternative to antibodies, as the number of antibody–receptor interactions is limited. In contrast, polymer ligands can be engineered in unlimited quantities through variations in molecular structure and composition, significantly expanding the range of parameters for fingerprint analysis. The employment of a diverse array of tags exhibiting distinct affinities allows for the analysis of fingerprints through an examination of the patterns in which macrophage cells bind to a collection of ligands.

#### 2.2.1. Trimannoside-PEG—Trapping Ligand

**Scheme 1:**
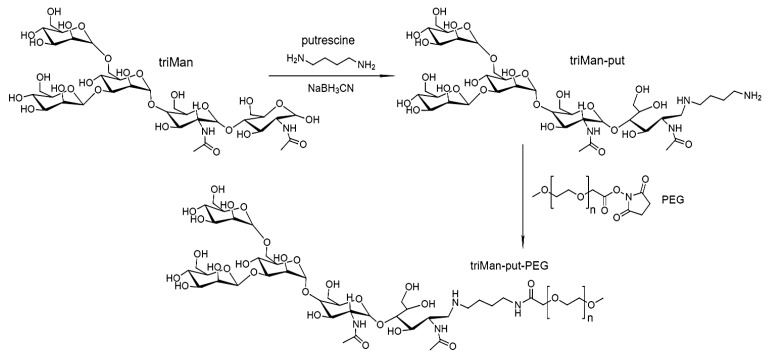
A scheme of synthesis of the trimannoside-PEG (triMan–PEG) trapping ligand.

The ligand synthesis scheme and FTIR spectra of the initial compounds and the product are presented in Scheme 1.

A mixture of 15 mg of triMan, 5 mg of putrescine and 4 mg of NaBH_3_CN was prepared and dissolved in 5 mL of a 0.2 mM hydrochloric acid solution. The mixture was then incubated at 45 °C for 6 h, followed by a purification process involving dialysis against water with a cut-off of 1 kDa. To obtained triMan-putrescine product, 55 mg of activated polyethylene glycol (PEG) with a molecular weight of 5 kDa was added to 10 mL of phosphate-buffered saline with a pH of 7.4. The resulting mixture was incubated for 12 h at 45 °C, after which it underwent another round of dialysis using water with a cut-off of 3.5 kDa.

#### 2.2.2. HPCD-PEI-triMan-FITC—Imaging Ligand

**Scheme 2:**
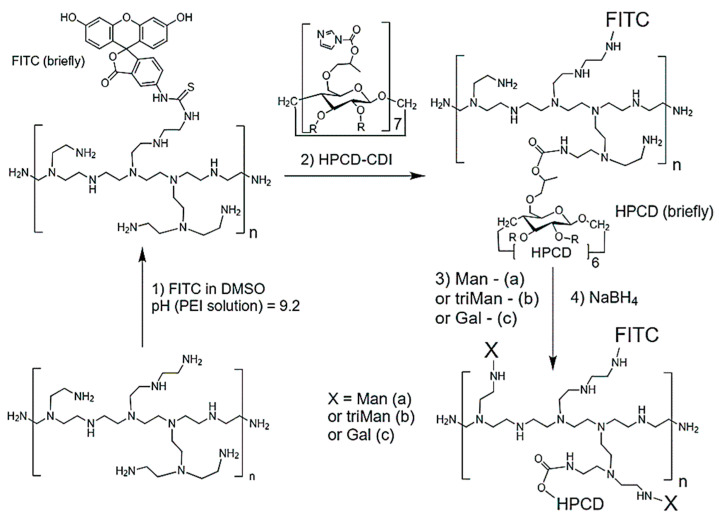
A scheme of synthesis of FITC-labeled HPCD-PEI-X “imaging ligand” for macrophage visualization.

The ligand synthesis scheme and FTIR spectra of the initial compounds and the product are presented in Scheme 2.

Firstly, activated HPCD was synthesized as described earlier [41,42] by the activation of HPCD OH-groups with carbonyldiimidazole (CDI) in DMSO.

Subsequently, FITC labeling of PEI was conducted. A solution of FITC (3 mg in 1 mL of DMSO) was gradually added to an aqueous solution of PEI (10 mL of 5 mg/mL in PBS) with stirring. The mixture was then incubated at 45 °C for two hours, followed by a 12 h dialysis process against PBS (with a cut-off of 1 kDa).

Following this, activated HPCD was introduced into the PEI-FITC solution in a dropwise manner, with a 2.5-fold molar excess relative to PEI chains. This mixture was further incubated at 45 °C for six hours in PBS, followed by another 12 h of dialysis against PBS with a cut-off ranging from 6 to 8 kDa.

Finally, a 5-fold molar excess of a trimannoside derivative (triMan) relative to PEI chains was added to the HPCD-PEI-FITC solution. Additionally, NaBH_3_CN was introduced in an amount that was 1.5 times the molar excess compared to triMan. The resulting mixture was subjected to incubation for 24 h at a pH of 5 and a temperature of 45 °C, followed by 12 additional hours of purification through dialysis using a membrane with a cut-off range of 6–8 kDa against distilled water.

#### 2.2.3. Characterization of Ligands

The final purification of the samples was performed by HPLC gel filtration in a Knauer chromatography system (Knauer, Berlin, Germany) on a Diasfer-110-C18 column (BioChemMack, Moscow, Russia). The concentration of the polymers was checked using CD spectroscopy (Jasco J-815 CD spectrometer, JASCO Corp., Tokyo, Japan).

All samples were freeze-dried for two days at −60 °C (Edwards 5, BOC Edwards, Burgess Hill, UK). The degree of mannosylation was calculated according to spectrophotometric titration of amino groups (before and after mannosylation) with 2,4,6-trinitrobenzenesulfonic acid.

Determination of the hydrodynamic diameter of the synthesized polymers was carried out by NTA (nanoparticle tracking analysis) using a Nanosight LM10-HS device (Nanosight, Amesbury, UK) [43].

The FTIR spectra of the samples in suspension were recorded using a Bruker Tensor 27 spectrometer equipped with a liquid-nitrogen-cooled MCT (cadmium–mercury telluride) detector. A total of 35 µL of samples was placed in a thermostatically controlled (22 or 37 °C) BioATR-II cell with a ZnSe element (Bruker, Bremen, Germany), and FTIR spectra in the range from 850 to 4000 cm^−1^ with a spectral resolution of 1 cm^−1^ were recorded. A total of 70 scans were accumulated and averaged for each spectrum. Spectral data were processed using the Bruker Opus 8.2.28 program (Bruker, Bremen, Germany).

Atomic force microscopy (AFM microscope NTEGRA II, NT-MDT Spectrum Instruments, Moscow, Russia) was used to study ligand particles’ formation, topology and size.

#### 2.2.4. Determination of Ligand Affinity to Model Mannose Receptor Concanavalin A

The process of complexation of concanavalin A with mannosylated ligands was carried out in PBS buffer (pH 7.4), which contained 0.5 M NaCl, 1 mM CaCl_2_ and 1 mM MnCl_2_. The concentration of concanavalin A was 1–10 mg/mL, and the ligand content ranged from 0.01-fold to 100-fold of the estimated dissociation constant (*K*_d_). The reaction was carried out at 37 °C for 30 min with continuous stirring. After the reaction, the FTIR spectra of the protein and the complexes were recorded, and the dissociation constants were determined using a standard protocol [44].

#### 2.2.5. Determination of Ligand Affinity to CD206+ by Macrophage Using Cytometry

Cytometry was performed on a BD FACSAria™ III Cell Sorter instrument (BD Bioscience, San Jose, CA, USA). The fluorescence of the FITC-labeled polymeric ligands captured by the CD206+ macrophage was analyzed. There was a change in the percentage of cells fluorescing in the FITC channel. Cells not incubated in the sample solution were evaluated as a control.

### 2.3. Bronchoalveolar Lavage Fluid (BALF) Isolation and Studies

#### 2.3.1. The Origin of BALF

BALF was procured on a voluntary basis from patients with primary ciliary dyskinesia (PCD), pneumonia and bronchial asthma at the Morozov Hospital. Five mg of *N*-acetylcysteine was added to BALF samples to dilute mucus, followed by washing them with PBS twice (4000× *g*, 5 min).

The composition of the BALF obtained from the patients was as follows. A patient with PCD (male, 5 years old): segmented neutrophils—72.7%, monocytes—12.2%, lymphocytes—3.8%, eosinophils—9.8%, macrophages—1.5%. A patient with pneumonia (female, 3 years old): segmented neutrophils—81%, monocytes—2.0%, lymphocytes—3.0%, macrophages—14%. A patient with bronchial asthma (female, 6 years old): segmented neutrophils—53.7%, macrophages—45.7%, lymphocytes—0.6%.

#### 2.3.2. CD206+ Macrophage Extraction

CD206+ macrophages were extracted from BALF using a complex methodology involving a sandwich approach. First, a sample of BALF was introduced into a trap constructed from a polymer containing trisaccharide residues, such as trimannoside polyethylene glycol (trimannoside-PEG). The sample was then incubated at 37 °C for a period of two hours, after which all unbound cells were carefully removed through washing with phosphate-buffered saline (PBS). Next, macrophages that had bound to the trap were visualized using a polymer ligand featuring fluorescein isothiocyanate (FITC), HPCD-PEI-triMan-FITC. This technique allows for the selective isolation of macrophages using ligands with dissociation constants (*K*_d_) in the range of 10^−6^–10^−7^ M, followed by their subsequent staining for further analysis.

#### 2.3.3. The Cultivation of a Reference Line for Activated CD206+ Macrophages

The acquisition of a reference line of activated macrophages was achieved through the use of a human monocyte cell line known as THP-1. These cells were obtained from the repository of cell lines maintained at Lomonosov Moscow State University. The THP-1 cells were cultivated in RPMI-1640 medium supplemented with GlutaMAX™ (Invitrogen, Carlsbad, CA, USA) and buffered using 10 mM HEPES at pH of 7.4, incorporating 10% heat-inactivated fetal bovine serum and 1% antifungal antibiotic. The cultivation was conducted at 37 °C and with 5% CO_2_. To generate macrophage-like cells, THP-1 at a concentration of 0.5 million cells per milliliter was treated with 100 nanomolar phorbol 12-myristate 13-acetate (PMA) for a duration of 72 h. After this period, the medium was exchanged, and the cells were allowed to continue culturing for an additional 96 h. Human dermal fibroblasts were obtained from skin samples collected from healthy donors undergoing abdominal surgery. In cases in which the donors were minors, informed consent was obtained from their parents or legal guardians. The study protocol was approved by the IRB00010587 Ethics Committee of the Lomonosov Moscow State University Medical Research and Education Center (Moscow, Russia). The cultures were maintained at 37 °C and with 5% CO_2_.

#### 2.3.4. Confirmation of Macrophage Polarization and CD206 Expression

Confirmation of macrophage polarization and CD206 expression was performed using confocal microscopy as well as cytometry assay. Non-specific binding sites were blocked using 10% solution of normal goat serum in PBS with 1% bovine serum albumin for 1 h at 22 °C. CD206 labeling was performed using anti-CD206 antibodies (ab64693, Abcam, Cambridge, MA, USA, 1:100) or rabbit polyclonal control IgG (910801, Biolegend, California, USA) as a control for 2 h at 22 °C and subsequently with goat–anti-rabbit antibody conjugated with Alexa594 (A11037, Molecular Probes, Invitrogen, Carlsbad, CA, USA, 1:1000). The nuclei were stained with DAPI. Samples were analyzed with a Leica DM6000B fluorescent microscope equipped with a Leica DFC 360FX camera (Leica Microsystems GmbH, Wetzlar, Germany). Most of these cells were CD206-positive according to immunocytochemical analysis. CD206-positive cells effectively phagocytosed predominantly high-affinity polymeric conjugates with trimannoside (trimannoside-PEG) or HPCD-PEI-triMan-FITC.

#### 2.3.5. FTIR-Mapping of the CD206+ Macrophages

The FTIR-mapping of the CD206+ macrophage from BALF was performed using a MICRAN-3 FTIR microscope equipped with a liquid-nitrogen-cooled MCT (mercury cadmium telluride) detector. Cell suspension from BALF (50 µL) was incubated with 20 µL of 10 mg/mL trimannoside-PEG solution in PBS for an hour at 37 °C. The unbound ligand was then removed by washing and centrifugation of the cells. A sample of 10 µL cells was applied to a metal plate and dried. After that, the FTIR spectra of c were recorded in the region of 100 × 100 µm^2^ in increments of 5 µm. The quantitative distribution of CD206 receptors was determined based on the integral intensity of the ν(C-O-C) peak at 1100–1000 cm^−1^, corresponding to the PEG.

### 2.4. Microbiology Experiments

#### 2.4.1. Bacterial Cells’ Cultivation

The *Escherichia coli* strains used in this study were as follows:

1. W3110 CGSC# 4474 (F-, λ-, IN(*rrnD-rrnE*)1, *rph*-1) [45];
2. BL21(DE3) CGSC#12504 (F-, *lon-11*, *Δ(ompT-nfrA*)885, Δ(*galM-ybhJ*)884, λDE3 [*lacI*, *lacUV5-T7 gene 1*, *ind1*, *sam7*, *nin5*], *Δ46*, [*mal+*]*K-12(λS)*, *hsdS10*) [46];
3. JM109 (F+ *Δ(lac-proAB)*, *recA1*, *endA1*, *gyrA96*, *thi-1*, *hsdR17*, *supE44*, *relA1*, [*F’*, *traD36*, *proAB*, *lacIq Z ΔM15*] [47];
4. MH-1 ΔrecA (F- *araD139*, *Δlac X74*, *galU*, *galK*, *hsdR2*, *rpsL ΔrecA srl*::*Tn10*) is a derivative of MH-1 that contains the *ΔrecA* deletion and the Tn10 insertion [48];
5. ATCC 25922 from the National Resource Center Russian Collection of Industrial Microorganisms, SIC “Kurchatov Institute”.

The *Lactobacillus fermentum* strains used in this study were from commercially available pharmaceutical Lactobacillus liquid concentrate.

The *Bacillus subtilis* (ATCC6633) strain used in this study was from the National Resource Center Russian Collection of Industrial Microorganisms, SIC “Kurchatov Institute”.

The cultures were cultivated for 18–20 h at 37 °C to a CFU/mL ≈ 4–7 × 10^8^ (determined by measuring A_600_ and by seeding them on Petri dishes) in the liquid nutrient LB medium (pH 7.2) with stirring at 120 rpm.

#### 2.4.2. Transformation of Bacterial Cells by pRed Plasmid

Plasmid pRed carrying red fluorescent protein gene [49] cloned into pQE30 (Qiagen, Hilden, Germany) was obtained from A.P. Savitsky (Institute of Biochemistry Russian Academy of Science, Moscow, Russia). Standard protocol for the transformation of plasmid DNA into chemically competent bacterial cells was used [50].

#### 2.4.3. Visualization of Petri Dishes with *E. coli* Expressing RFP

Fluorescent images of a Petri dish with *E. coli* cells (10^7^ CFU/0.5 mL placed onto 20 mL of agar medium) after 18 h of incubation at 37 °C (and a subsequent two-day maturation of RFP at 4 °C) were obtained with a UVP BioSpectrum imaging system (BioSpectrum; UVP, Upland, CA, USA). λ_exci,max_ = 480 nm. λ_emi_ = 485–655 nm.

#### 2.4.4. Fluorescence Spectroscopy

The fluorescence emission spectra of bacterial cells expressing RFP and methylene blue (MB)-labelled *Lactobacillus* suspensions (4 × 10^8^ CFU/mL) were recorded on a Varian Cary Eclipse fluorescence spectrometer (Agilent Technologies, Palo Alto, CA, USA). For RFP, λ_exci_ was set at 520 nm. For MB, λ_exci_ was set at 630 nm. T = 37 °C.

### 2.5. Incubation of Macrophages with E. coli

#### 2.5.1. Kinetic Curves of *E. coli* Phagocytosis

Incubations of macrophages with *E. coli* in the absence and presence of *Lactobacilli* competitor were performed in 96-well plates or sterile 2 mL Eppendorf tubes. The macrophage density was 10^6^ cells per well plate. The bacterial density was 5 × 10^7^ for all strains. The following conditions were used for the LB medium/PBS: 50:50 *v*:*v*, pH 7.4 and 0.01 M. T = 37 °C. Kinetic curves of *E. coli* phagocytosis by CD206+ macrophages from the BALF were measured fluorometrically: λ_exci,max_ = 520 nm and λ_emi_ = 550, 580 nm. A SpectraMax M5 device (Downingtown, PA, USA) in a Costar black/clear-bottom tablet (96 wells) was used.

#### 2.5.2. CLSM Visualization of Phagocytosis

Confocal laser scanning microscopy (CLSM) images of CD206-positive macrophages derived from the BALF phagocytes (and reference macrophage cell line derived from human THP-1 monocytes) of *Escherichia coli* in the absence and presence of *Lactobacilli* competitor were obtained using an Olympus FluoView FV1000 (Olympus, Tokyo, Japan) confocal laser scanning microscope equipped with both a spectral version scan unit with emission detectors and a transmitted light detector. CLSM is based on the Olympus IX81 motorized inverted microscope (Olympus, Tokyo, Japan). The Olympus UPLSAPO 40X NA 0.90 objective lens was used for the measurements (Olympus, Tokyo, Japan). Laser power, sampling speed and averaging were the same for all image acquisitions. The scan area was 80 × 80 µm^2^. Macrophage nuclei were stained with DAPI (λ_ex,max_ = 360 nm; λ_emi_ = 400–500 nm) on fluorescent microscope. Other channels were registered using CLSM technique: macrophages were stained with FITC anti-CD206 ligand (HPCD-PEI1.8-triMan-FITC)—green channel (λ_ex,max_ = 488 nm; λ_emi_ = 505–555 nm). *E. coli* cells were stained with RFP—red channel (λ_ex,max_ = 559 nm; λ_emi_ = 575–625 nm). *Lactobacilli* cells were stained with methylene blue (MB)—cyan channel (λ_ex,max_ = 635 nm; λ_emi_ = 650–750 nm). The signals were adjusted to the linear range of the detectors. Olympus FV10 ASW 1.7 software was used for the acquisition of the images.

### 2.6. Profiling of the Phagocytic Activity of Macrophages from BALF in Relation to the Clinical Diagnosis

The experiment was carried out similarly to the one described above in Section 2.5.1: various bacterial cells (*E. coli* strain W3110, *L. fermentum* strain and *B. subtilis* ATCC6633 strain) were incubated with macrophages from BALF. The degree of binding of bacterial cells by macrophages was determined after 2 h of incubation in the presence or absence of mannan (a competitive ligand for CD206 receptors). The set of binding percentages is the profile of phagocytic activity of macrophages, which we compared with the severity of the disease and the diagnosis.

## 3. Results and Discussion

### 3.1. Work Design

The aim of this study is to develop a method for characterizing activated macrophages based on variations in phagocytic activity across different cellular types, which could be possibly applied as a valuable diagnostic tool for certain pulmonary diseases. Here, we have developed trimannoside-based polymeric ligands to extract macrophages from the BALF samples of patients with PCD for the subsequent study of their phagocytic activity, including visualization with confocal fluorescence microscopy (CLSM). Five distinct strain types of *E. coli* cells (including in the presence of the *Lactobacillus* competitor) were tested to find the differences in the macrophages phagocytic activity depending on the surface morphology of the bacterial strain.

The following objectives were fulfilled:

1. The cultivation of the *E. coli* bacterial strains MH-1, JM109, BL21, W3110, and ATCC25922, the *L. fermentum* strain and the *B. subtilis* ATCC6633 strain differed in origin, surface morphology and antibiotic resistance. Each bacterial strain expresses red fluorescent protein (RFP) to visualize phagocytosis by fluorescent methods;
2. A spectral analysis of the fluorescent properties of the cultivated strains, along with the acquisition of confocal and electron microscopic images, was conducted;
3. By employing the highly specific polymer ligands we have developed, which exhibit a strong affinity for activated macrophages bearing the CD206 mannose receptor, we are able to isolate CD206+ macrophages from the BALF using highly specific mannose-functionalized “trapping ligands”;
4. An investigation of the phagocytosis kinetics of *E. coli* strains by macrophages, depending on the strain phenotype in the presence or absence of a *Lactobacilli* competitor, was conducted. A comparison was made with the reference model macrophage cell culture;
5. A visualization of the *E. coli* bacteria’s phagocytosis by macrophages, depending on the strain specificity and the presence of *Lactobacilli* competitors, was performed;
6. Profiling of the phagocytic activity of macrophages from BALF in relation to the clinical diagnosis using various bacterial cells (the *E. coli* strain W3110, the *L. fermentum* strain and the *B. subtilis* ATCC6633 strain) was conducted.

### 3.2. The Characterization of Escherichia coli Strains

The bacterial strains of *Escherichia coli* MH-1 [48], JM109 [47], BL21 [46], W3110 [45] and ATCC25922 [51,52] with expressed red fluorescent protein (RFP) exhibit differences in their surface morphology, origin and susceptibility to antibiotics (as summarized in Table 1). All the strains have a rod-like shape, which is characteristic of *E. coli*, with dimensions ranging from 0.5 to 1 µm in width and from one to three µm in length (according to TEM).

With regard to the strain JM109, the presence of both protein pili and flagella is observed. In contrast, the strain MH-1 is characterized mainly by the presence of flagella. The average length of these flagella ranges between 2.5 and 4 μm, while the length of fimbriae is in the range of 0.3 to 1 μm (according to TEM). The findings obtained in this study corroborate the existing literature data regarding the structural characteristics of the strain JM109. The genetically engineered strain MH-1 proved to be the most sensitive to the antibiotic levofloxacin, with an MIC of 0.10 ± 0.02 μg/mL. The colonies produced by MH-1 were small, and the bacteria themselves were relatively small, measuring 0.6 by 1.5 μm (according to TEM). The MH1 strain exhibits a distinctive feature in the form of pili, which endows them with enhanced mobility. In contrast, the genetically modified strain JM109 exhibited significantly greater resistance (than MH-1) to the effects of the antibiotic, with an MIC value of 1.0 ± 0.1 μg/mL. This significant difference in MIC values between the two genetically modified strains may be attributed to differences in the surface structures of their bacterial cells. The JM109 strain exhibits fimbria protein structures atop its cell wall, which serves as an additional barrier against the entry of antibacterial agents. These colonies are compact, with the bacteria themselves being relatively small but larger than those of MH-1, measuring 0.6 by 2 μm (Table 1). The JM109 and MH-1 strains exhibit a relatively straightforward transformation process, resulting in the formation of vivid pink colonies. In the fluorescent channel, distinct red dots corresponding to individual cells measuring one to two μm in size can be observed using CLSM. The JM109 strain exhibits robust growth and is amenable to being efficiently transformed through various techniques. Due to its lack of the *E. coli* restriction system and the presence of RecA (RecA is a 38 kDa protein essential for the repair and maintenance of DNA in bacteria), the undesired restriction of cloned DNA or recombination with host chromosomal DNA is excluded. The mutation of endonuclease A results in an enhancement in both the yield and purity of isolated plasmid DNA.

*E. coli* BL21(DE3), a strain commonly employed for protein production, exhibits a unique combination of attributes that enables the overexpression of heterologous proteins. This strain is derived from the B lineage of *E. coli*. The BL-21 strain demonstrates a remarkable resistance to antibiotics, with a minimum inhibitory concentration (MIC) ranging from 1.5 to 2 μg/mL. The colonies formed by this strain appear larger, and the bacteria themselves are relatively small, approximately 0.5 μm × 1.2 μm (as observed by TEM). This strain is also easily transformed with a red plasmid, leading to the development of large, vivid pink colonies. Furthermore, in the fluorescent channel using CLSM, bright red rod-like structures corresponding to individual cells up to two to three μm in length can be discerned.

*E. coli* W3110 and MH-1 strains exhibit pronounced pili. *E. coli* ATCC25922 was obtained from a collection of microorganisms, and its cell colonies exhibit a filamentous morphology, as confirmed by TEM and CLSM analysis. These strains also exhibit the presence of mucus.

Wild strains are characterized by their high antibiotic resistance. For the ATCC25922 wild strain, the minimum inhibitory concentration (MIC) is 2.4 ± 0.3 μg/mL, while for the W31110 strain, it is approximately 1.8 ± 0.2 μg/mL. These wild strains form large pink/beige colonies, with bacteria measuring approximately 0.6–0.8 μm in width and 2–3 μm in length, as observed through TEM analysis. These cells exhibit a decreased level of fluorescence, with fewer glowing red dots visible in the fluorescent images compared to other strains.

### 3.3. Fluorescence Properties of Escherichia coli Strains Expressing RFP and Lactobacilli Labelled with MB

Variations in the cellular surface architecture may give rise to diverse phagocytic behaviors and, in turn, for macrophages with different polarization and phenotypes, the determined indices of relative phagocytic activity for a set of model bacterial cells will be individual and determine a specific profile—a histogram of their phagocytic activity. This provides us with a tool for the quantitative characterization of macrophages’ subpopulations of heterogeneous BALF samples.

**Figure 1:**
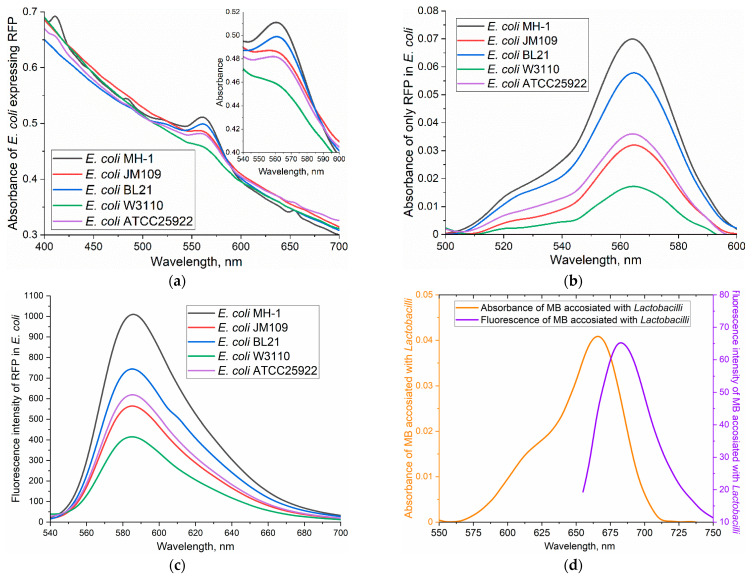
(**a**) Molecular absorption spectra of bacterial suspensions (4 × 10^8^ CFU/mL) of *E. coli* MH-1, JM109, BL21, W3110 and ATCC25922 expressing red fluorescent protein (RFP). (**b**) Molecular absorption spectra of only RFP in the same bacterial suspensions. (**c**) Fluorescent spectra of the same bacterial suspensions (4 × 10^8^ CFU/mL) of *E. coli* MH-1, JM109, BL21, W3110 and ATCC25922 at λ_exci_ of 520 nm. (**d**) Molecular absorption and fluorescent spectra of *Lactobacilli* cells stained with methylene blue (MB) at λ_exci_ of 630 nm and T = 37 °C.

To investigate phagocytosis mediated by macrophages, we have developed engineered strains of *E. coli* that express red fluorescent protein (RFP), which will be used in subsequent studies employing fluorescence and confocal microscopy techniques. Figure 1 presents the molecular absorption and fluorescent spectra of five strains of *E. coli*, namely MH-1, JM109, BL21, W3110 and ATCC25922, each with a concentration of 4 × 10^8^ CFU/mL. The absorption spectrum of the *E. coli* suspension encompasses two primary components: (i) a declining curve, which corresponds to the scattering of light by bacterial cells (it serves as an ancillary means of monitoring the cell count in samples); and (ii) a peak of RFP absorption ranging from 540 to 590 nm, with a maximum value at 560 nm. To record the fluorescent spectrum, we employed an excitation wavelength of 520 nm for all strains. The maximum emission is at 590 nm.

Additionally, we stained *Lactobacilli* cells with methylene blue (MB, Figure 1d), providing a possibility to follow them upon the competition with *E. coli* during phagocytosis. The absorption spectrum of the *Lactobacilli* suspension also encompasses two primary components: (i) a declining curve, which corresponds to the scattering of light by bacterial cells; and (ii) a peak of MB absorption ranging from 590 to 710 nm, with a maximum value at 660 nm. To record the fluorescent spectrum, we employed an excitation wavelength of 630 nm for the *Lactobacilli* suspension. The maximum emission was observed at 685 nm. Thus, we could observe each component in a confocal microscope in the presence of the other components, which is analytically significant.

### 3.4. Highly Specific Ligands for the Isolation and Characterization of CD206+ Macrophages from Biological Fluids

To extract and characterize CD206+ macrophages from BALF and determine their functional properties and polarization status, we have developed a comprehensive set of biosensors based on specific polymer ligands targeting CD206 receptors on macrophages.

**Figure 2:**
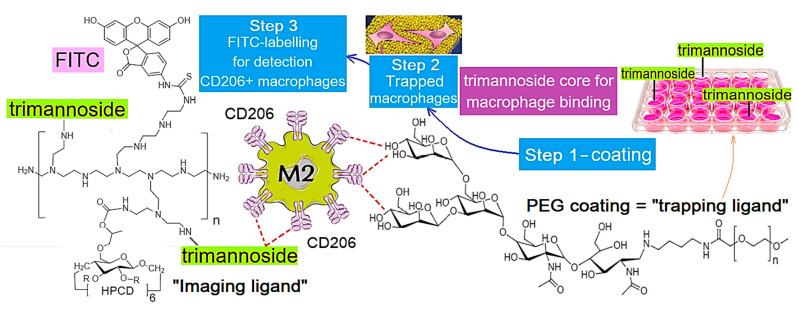
A schematic representation of the “sandwich” approach for the isolation and staining of CD206+ macrophages in biological fluids employing the use of high-affinity trimannoside ligands as trapping and imaging tools. The trapping ligand is triMan-PEG-PEG, and imaging ligand is HPCD-PEI-triMan-FITC (Scheme 1 and Scheme 2).

Here, we used two categories of ligands (Table 2, Figure 2): a “trapping ligand” and an “imaging ligand”. The synthesis schemes and infrared spectra of these ligands are provided in Scheme 1 and Scheme 2. The first category comprises high-affinity ligands to CD206 receptors with trimannoside residues (trimannoside-PEG) that serve as “traps” for macrophages (Table 2). The second category comprises “imaging” ligands equipped with a fluorescent label (FITC), which serve to visualize and study macrophages that have been captured. For this purpose, we employed a specific ligand, cyclodextrin-polyethyleneimine-trimannoside conjugated with FITC (HPCD-PEI-triMan-FITC) (Table 2).

The successful synthesis of triMan-PEG is evident from the presence of a prominent peak at 1090 cm^−1^ for the C-O-C bond in the Fourier transform infrared (FTIR) spectra of the products (Figure S1). Additionally, a minor peak at 1030 cm^−1^ is observed, corresponding to the ν(C-O) vibrations in carbohydrates. The formation of amide bonds is also supported by the appearance of peaks at 1660 cm^−1^ for ν(C-O) and 1580 cm^−1^ for δ(N-H), replacing the peak typically observed at 1710 cm^−1^ for activated PEG ethers (Figure S1). Figure S2 shows the infrared spectra of fluorescent markers derived from polyethyleneimine (PEI) with modifications that incorporate linear galactose, cyclic mannose and trimannoside residue. The spectra reveal clear vibrational bands of CH2 groups in the PEI region, ranging from 2980 to 2800 cm^−1^. The characteristic peaks at 1580 and 1450 cm^−1^ correspond to the stretching vibrations of C-C in the fluorescein isothiocyanate (FITC) label. The peaks in the range of 1200 to 1000 cm^−1^ are associated with the vibrations of C-N in the PEI molecule and C-O-C bonds in the saccharide components.

Polymers form particles with a hydrodynamic size of about 100 nm with a weakly positive charge in the case of trimannoside-PEG and with a high positive zeta-potential in the case of HPCD-PEI-triMan-FITC. The presented polymers are characterized by a high affinity (with K_dis_ 10^−7^ M) to the model mannose CD206 receptor (ConA), which served as a reference for the characterization of ligands, since cells are always different and the protein model receptor is a good standard testing agent. Reference line CD206+macrophage cells in the M1 state (derived from THP-1 in vitro) also absorb mannosylated particles, which confirms the selectivity of imaging and trapping ligands to CD206 receptors of macrophages (Table 2).

The process of isolating and visualizing macrophages can be realized as a “sandwich method” (Figure 2). (i) A sample of BALF is applied to a “trap” composed of a polymer containing trimannoside residues (triMan-PEG) (incubation at 37 °C for 2 h). Unbound cells are subsequently removed by washing. (ii) Macrophages that have bound to the “trap” are visualized using a polymeric ligand containing FITC. This technology enables, on the one hand, the selective isolation of macrophages using high-affinity ligands (*K*_dis_ for the model ConA-ligand of 10^−7^ M (Table 2)), and, on the other hand, their subsequent “imaging” for further analyses (Figure 2).

### 3.5. The Isolation and Characterization of CD206+ Macrophages from BALF

**Figure 3:**
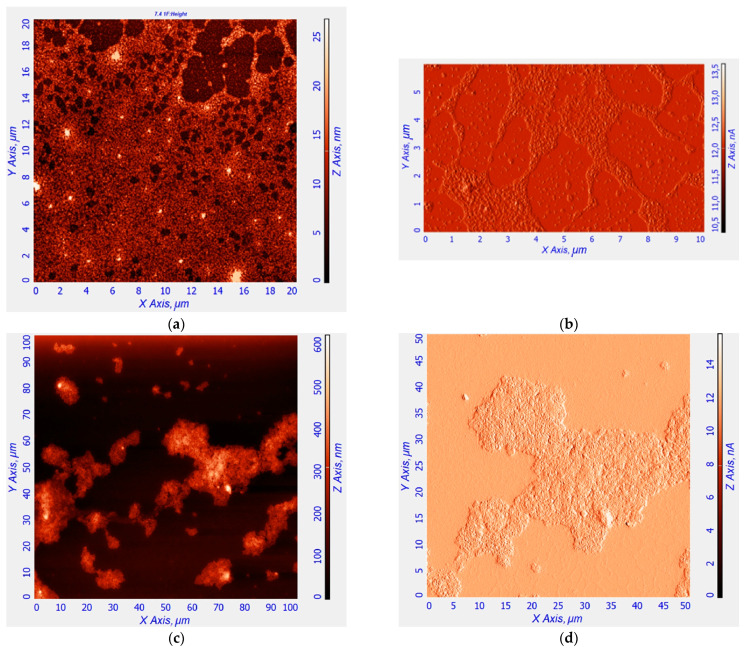
(**a**) AFM image of a “trap polymer” film (trimannoside-PEG) deposited onto a freshly cleaned mica surface. (**b**) An enlarged image. (**a**) A magnitude signal. (**c**) AFM image of macrophages from the BALF deposited onto freshly cleaned mica surface. (**d**) Increased AFM image; (**c**) magnitude signal.

To isolate and characterize the status of activated macrophages from BALF, the macrophages, which express the CD206 receptor, are trapped in a polymer film based on trimannoside-PEG as described above. The resulting polymer trap film was imaged using atomic force microscopy (AFM), as shown in Figure 3a,b. The average height of the polymeric film is between 10 and 20 nm. Spherical structures, approximately 100 nm in diameter and 50 nm in height, can also be seen.

The macrophages from the BALF were captured in the trap, and their AFM image is shown in Figure 3c,d. The size of these macrophages varies between 8 and 15 µm, and their average height is approximately 300 to 500 nm. An enlarged view of a group of macrophages in the shape of a cat is shown in the 2D figure.

**Figure 4:**
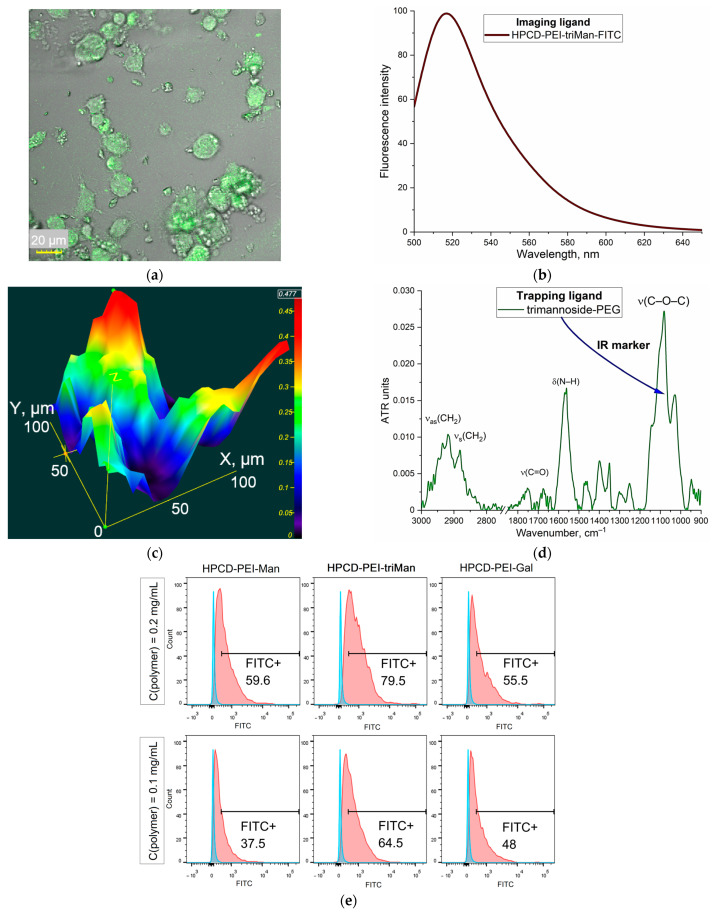
(**a**) CLSM images of CD206+ macrophages stained with HPCD-PEI-triMan-FITC imaging ligand. λ_exci_ = 488 nm. λ_emi_ = 505–600 nm. (**b**) Fluorescent spectra of HPCD-PEI-triMan-FITC imaging ligand (10 µg/mL) at λ_exci_ of 480 nm. (**c**) A map of the spatial distribution of the integrated signal intensity in the FTIR spectra of the triMan-PEG “visualization ligand” associated with CD206+ macrophages revealing a pattern characterized by a peak ν(C–O–C) at 1100–1000 cm^−1^. The regions marked with red correspond to areas with a high concentration of the ligand, which in turn is associated with the expression of the CD206 receptor, while blue indicates the absence of receptors. (**d**) FTIR spectrum of “trapping ligand” triMan-PEG. PBS (pH 7.4, 0.01 M). T = 37 °C. (**e**) Histograms of CD206+ macrophage distribution by FITC fluorescence intensity when stained with HPCD-PEI-triMan-FITC imaging ligand (compared with medium-affinity mannose ligand and low-affinity galactose ligand) according to flow cytometry data.

The data obtained by AFM are corroborated by CLSM (Figure 4a), in which individual macrophages exhibit fluorescence in the FITC channel, which is a result of the interaction with the HPCD-PEI-triMan-FITC ligand (Figure 4b). The dimensions of macrophage cells are approximately 20 µm.

A further verification of the expression of the CD206 receptor on isolated macrophages was achieved through the use of FTIR microscopy (Figure 4c,d). The spatial distribution map of the integrated signal intensity in FTIR spectra associated with trimannoside-PEG ligands bound to the CD206+ macrophages reveals a pattern characterized by a peak in the ν(C-O-C) region at 1100–1000 cm^−1^. This pattern demonstrates areas (in red) with a higher level of CD206 receptor expression, whereas blue indicates the absence of CD206 receptors. The red peaks on this map have a diameter of approximately 15–20 µm, which precisely corresponds to the size of a macrophage cell. IR mapping allows us to ascertain that the distribution of CD206 receptors on activated macrophages is not clustered but is rather more uniform.

Macrophage cells with CD206 expression effectively phagocytosed mainly high-affinity polymer conjugates with the trimannoside label—imaging ligand HPCD-PEI-triMan. To a lesser extent, macrophages also captured conjugates with linear mannose or galactose labels, but this was not specific and was due to polymer adhesion (Figure 4e). A cytometry assay showed that 79.5% of macrophage-like cells were FITC-positive after adding HPCD-PEI-triMan, 59.6% were FITC-positive after adding HPCD-PEI-Man and 55.5% were FITC-positive after adding HPCD-PEI-Gal at a concentration of 0.2 mg/mL (Figure 4e). After reducing the polymer concentration twice, the uptake of FITC was 64.5%, 37.5% and 48.2%, respectively (Figure 4e). Human dermal fibroblasts showed a very low level of CD206 expression and polymer uptake according to our previous data [33].

Thus, we have successfully isolated CD206+ macrophages from the BALF samples of patients with respiratory diseases. Further, we aimed to determine the phagocytic profile of these BALF macrophages with respect to *E. coli* in comparison with human CD206+ macrophages (M1 polarization) derived from THP-1 monocyte cultures used as a reference model.

### 3.6. Binding of CD206+ Macrophages Derived from BALF to Escherichia coli Cells

**Figure 5:**
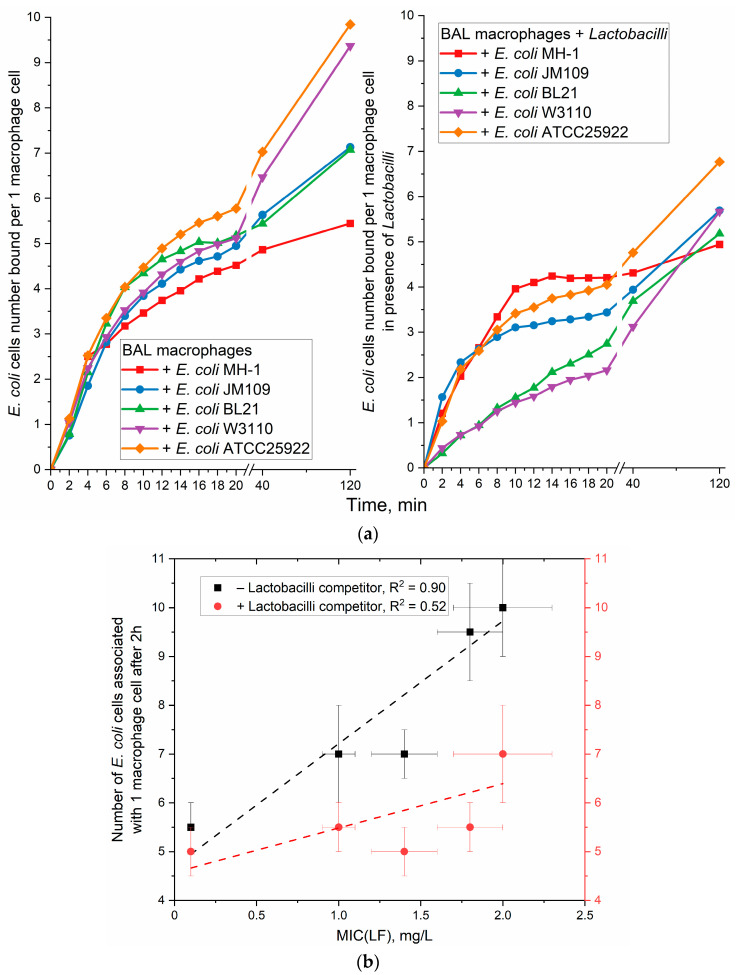
(**a**) Kinetic curves of *E. coli* phagocytosis by CD206+ macrophages from the BALF in the absence and presence of *Lactobacilli* competitor. Kinetic curves were recorded by changing the fluorescence of the red protein as a percentage of the initial one. (**b**) The correlation between the number of bacteria interacting with macrophages and MIC values for levofloxacin (LF). The dotted line shows the trend lines and confidence coefficients. The macrophage density was 10^6^ cells per well plate. The bacterial density was 5 × 10^7^ for all strains. LB medium/PBS (50:50 *v*:*v*, pH 7.4, 0.01 M). T = 37 °C.

We consider phagocytic activity towards bacteria as an important functional parameter that characterizes the polarization state of macrophages and, correspondingly, the macrophages’ functional properties. Here, we used five strains of model *E. coli* bacteria to test the phagocytic activity of the CD206+ macrophages. Figure 5a presents the kinetic curves of *E. coli* interaction (binding) by CD206+ macrophages isolated from BALF, both in the absence and in the presence of a *Lactobacilli* competitor. This yields additional quantitative parameters to characterize the functional properties and features of the macrophages under study. We measured the kinetics of bacterial cell interaction macrophages using short observation times (up to 2 h). Kinetic curves were recorded by changing the fluorescence of the red-protein-containing cells as a percentage of the initial intensity.

We observed that the phagocytosis degree is correlated with bacterial resistance to antibiotics (MIC parameter, Figure 5b). Meanwhile, *Lactobacilli* could hinder *E. coli* from macrophage phagocytosis due to the reduced availability of endocytic sites. Table 3 provides quantitative data on *E. coli* bacterial binding with macrophages, including the total number of captured bacteria after 2 h of incubation and the *E. coli* binding rate. The first parameter reflects the efficiency of phagocytic defense against pathogens, which is highest for wild-type strains of *E. coli* (W3110 and ATCC25922). In the absence of *Lactobacilli*, the rate of *E. coli* binding to macrophages was five to ten bacterial cells per Mph cell (per 2 h), while in the presence of the competitor, it decreased by 1.5–2 times. It should be noted that *Lactobacilli* are taken up by Mph to a lesser extent at a rate of about three bacterial cells per one macrophage cell in 2 h.

The initial rate of *E. coli* binding was highest for *E. coli* strains MH-1 and ATCC25922. This phenomenon can be attributed to a number of factors, including the increased surface villosity of bacterial cells due to the presence of pili and fimbriae (Table 1). An important observation is the relationship between the level of interaction between bacteria and macrophages (phagocytosis) and antibiotic MIC values (shown in Table 1)—the good correlation (with R^2^ = 0.90 for a linear approximation) of these parameters is observed (Figure 5b). This means that more antibiotic-resistant strains, which are often more pathogenic, are more efficiently absorbed by macrophages. However, when a competitor (*Lactobacilli*) is present, this correlation is lost. Apparently, the effectiveness of phagocytosis depends on the presence or absence of a competitor. The comparative indexes of phagocytic activity to a set of model bacterial cells and the changes in these indexes in the presence of a *Lactobacilli* competitor can be considered as a parameter for the assessing of the alveolar macrophages’ subpopulation profile for subsequently assessing the status of inflammation and determining dangerous conditions in the case of lung inflammatory diseases.

The presence of fimbriae and pili on the surface of bacteria contributes to a significant enhancement in the capacity of these microorganisms to be bound by macrophages, potentially reaching up to 50% of the total number of cells. The strains of *E. coli* MH-1 and JM109 were characterized by ζ-potential values of −37 ± 2 mV and −35.5 ± 2.5 mV, respectively, consistent with the literature data for other *E. coli* strains [53,54,55]. Fimbriae do not significantly alter the ζ-potential values. But fimbriae serve as a shield against the charge of the bacterial cells and contribute to the enhancement of macrophage phagocytosis.

To conclude, it is worth noting that by employing the macrophage trap method, it became possible to extract activated CD206+ macrophages from the BALF of patients with PCD. These macrophages actively phagocytose bacterial cells to varying degrees. The BALF macrophages were compared to model CD206 cell lines derived from THP-1 human monocytes that exhibited a robust phagocytic capacity [32,44]. Conversely, a negative control was employed, consisting of CD206 cells devoid of pronounced phagocytic activity.

So, the kinetic experiment yielded the impact of both the adhesion of bacterial cells to the surface of macrophages and the cells’ uptake. However, the uptake of cells during a one-hour experiment might be relatively lower. Therefore, phagocytosis after 4 h of incubation of macrophages with bacterial cells was determined using confocal microscopy.

### 3.7. Confocal Laser Scanning Microscopy for Visualization of CD206+ Macrophages from BALF Phagocyted Escherichia coli Cells

#### 3.7.1. Comparison of the Phagocytosis for BALF Macrophages in Comparison with Model CD206+ Macrophages’ Cell Lines

**Figure 6:**
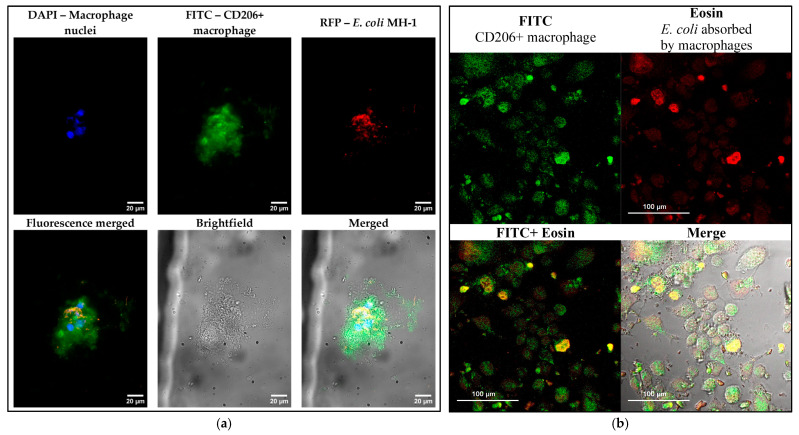
(**a**) Confocal laser scanning microscopy images of CD206+ macrophages derived from BALF after 4 h of incubation with *E. coli* MH-1 cells in the presence of *Lactobacilli* competitor cells. Macrophage nuclei were stained with DAPI (λ_ex,max_ = 360 nm; λ_emi_ = 400–500 nm); macrophages were stained with FITC anti-CD206 ligand (HPCD-PEI1.8-triMan-FITC)—green channel (λ_ex,max_ = 488 nm; λ_emi_ = 505–555 nm). *E. coli* cells were stained RFP—red channel (λ_ex,max_ = 559 nm; λ_emi_ = 575–625 nm). (**b**) Confocal laser scanning microscopy images of CD206+ macrophages derived from model human THP-1 monocytes after 4 h of incubation with *E. coli* MH-1 cells in the presence of *Lactobacilli* cells. Macrophages were stained with FITC anti-CD206 ligand (HPCD-PEI1.8-triMan-FITC)—green channel (λ_ex,max_ = 488 nm; λ_emi_ = 500–520 nm). *E. coli* cells were stained with eosin (covalent)—red channel (λ_ex,max_ = 515 nm; λ_emi_ = 530–600 nm).

Figure 6a presents an example of confocal laser scanning microscopy (CLSM) images of CD206-positive macrophages derived from the BALF phagocytes of *E. coli* in the presence of *Lactobacilli* competitor cells. The macrophage nuclei were stained with 4′,6-diamidino-2-phenylindole (DAPI), while the CD206 receptors were labeled with FITC on the polymer. *E. coli* bacteria exhibit fluorescence due to the expression of RFP. Within a period of 4 h, the macrophages captured a substantial number of bacterial cells (Figure 6a). These findings suggest that macrophages from the BALF exhibit a remarkable phagocytic capacity against model microorganisms, and it can be registered after 4 h of incubation.

To profile the macrophages’ characteristics and the phagocytosis activity, in addition, human CD206+ macrophages (M1 polarization) derived from THP-1 monocyte cultures were used as a reference model. The yellow spots correspond to macrophages that have absorbed *E. coli* (the overlapping green and red colors). The model macrophages (18 ± 5 µm) exhibit a remarkably narrower distribution in terms of size and morphology (a spherical and uniform shape) when compared to cells isolated from the BALF of the patient (20 ± 12 µm, with a disordered and heterogeneous form). The phagocytic activity exhibited by model M1 macrophages was lower than that demonstrated by the activated macrophages obtained from a patient with PCD.

#### 3.7.2. Phagocytic Activity of CD206+ Macrophages from BALF Towards Different Strains of *E. coli* Cells

**Figure 7:**
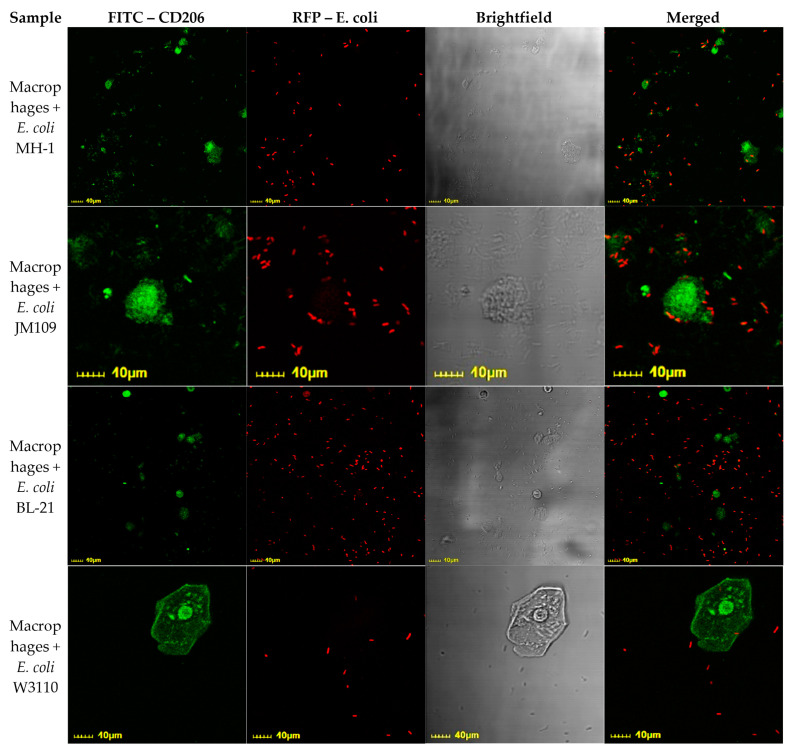
Confocal laser scanning microscopy images of CD206+ macrophages derived from BALF after 4 h of incubation with different *E. coli* cells. Macrophages were stained with FITC anti-CD206 ligand (HPCD-PEI1.8-triMan-FITC)—green channel (λ_ex,max_ = 488 nm; λ_emi_ = 505–555 nm). *E. coli* cells were stained RFP—red channel (λ_ex,max_ = 559 nm; λ_emi_ = 575–625 nm).

The macrophages exhibit varying degrees of phagocytic activity towards different strains of *E. coli* cells, as depicted in Figure 7, which presents CLSM images of CD206+ macrophages from BALF after a 4 h co-incubation with diverse *E. coli* strains. The degree of phagocytosis is significantly influenced by the morphological features of the bacterial surface. Relative indexes of the phagocytosis degree to the diverse standard *bacterial* strains (*E. coli*) can be considered as a parameter for the fingerprint analysis for the evaluation of the Mph subpopulation profile in BALF. Specifically, the highest value of the number of phagocytized bacteria is achieved for the strain JM109, characterized by the pronounced presence of protein fimbriae and pili that actively adhere to the macrophage surface before being engulfed. The phagocytic activity of macrophages against the MH-1 strain is less pronounced. Other strains display a lower phagocytic efficiency. Across all strains, the average number of phagocytized bacteria per macrophage ranges between five and six.

Figure S3 presents CLSM images of CD206+ Mph obtained from BALF after a 4 h co-incubation with diverse *E. coli* strains in the presence of *Lactobacilli* competitor cells. In this case, the phagocytic capacity of Mph against the MH-1 and JM109 strains of *E. coli* increases to up to 50 and 30 phagocytized bacteria per macrophage, correspondingly. This may be explained by the tendency of Mph phagocytosys to activate in the presence of *Lactobacilli* cells.

If we compare these data with the results obtained using the fluorescent binding analysis (Figure 5), one can see that by using fluorescence spectroscopy in the suspension, we measured the combined effect of phagocytosis and adsorption, with the latter mechanism making a more significant contribution. However, during the preparation of the samples for CLSM, all weakly bound bacteria were removed, and thus, we identified only phagocytized bacteria using microscopy.

Therefore, in the BALF sample of the patient with PCD:

(1) The **binding ability** (according to fluorimetry) of macrophages from the BALF against *E. coli* strains increases in the order of MH-1 < JM109 ≈ BL-21 < W3110 <≈ ATCC25922, which is correlated with the resistance of bacteria, expressed in terms of the minimum inhibitory concentration of the antibiotics;
(2) The **phagocytic ability** (according to CLSM) of macrophages from the BALF against *E. coli* strains increases in the order of BL-21 ≈ W3110 < ATCC25922 << MH-1 ≈ JM109, which is correlated with the presence of pili and/or fimbriae in bacteria.

### 3.8. Relationship Between Diagnosis and Phagocytic Activity of Macrophages

**Figure 8:**
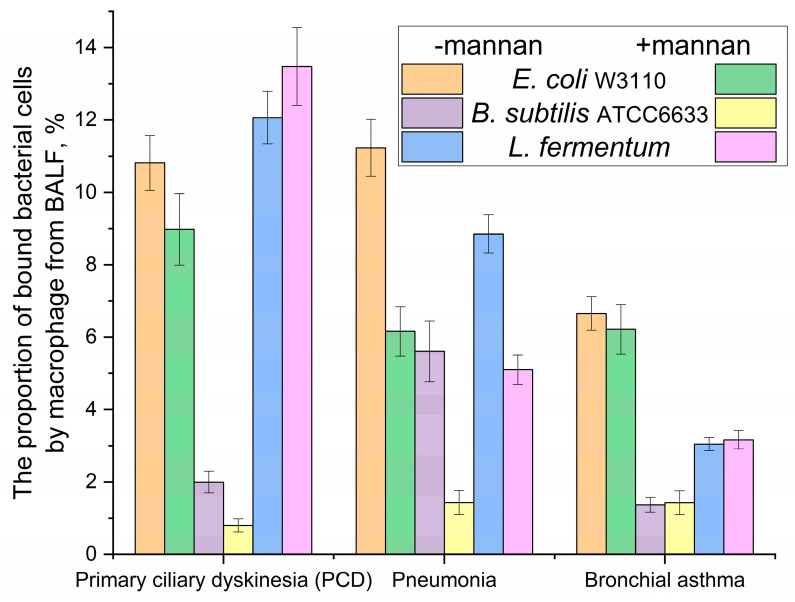
Correlation analysis of the phagocytic activity of CD206+ macrophages in BALF against different bacterial cells, depending on the presence or absence of mannan, a competitive ligand for CD206 receptors.

The relationship between the patients’ diagnostic results and the phagocytic activity of macrophages was analyzed (Figure 8). The proportion of bacterial cells associated with CD206 + macrophages from the BALF was affected by the presence of mannan, a competitive ligand for CD206 mannose receptors, which play a key role in bacterial phagocytosis by macrophages. In the absence of mannan, *E. coli* W3110 exhibits a relatively high level of bacterial phagocytosis, approximately 10%, for all of the patient categories. However, the presence of mannan (the CD 206 concurrent ligand) strongly inhibits bacterial binding by macrophages in cases of pneumonia, in which macrophages overexpress CD206 receptors.

*Bacillus subtilis*, by its nature, is less efficiently phagocytized compared to other bacteria. However, in the context of pneumonia, macrophages exhibit an increased phagocytic response towards these bacteria, which is also specifically suppressed by mannan.

*Lactobacillus fermentum* exhibits low binding in cases of asthma or pneumonia. Conversely, the markedly higher binding observed in the PCD group could suggest a potential divergence in the sensitivity of macrophages towards this specific type of bacteria, potentially due to an impairment in the functioning of immune system cells.

The data presented herein reveal a complex interplay between diagnostic markers and the phagocytic activity of macrophages. Notably, in acute inflammatory conditions such as pneumonia and acute bronchitis, a heightened level of phagocytosis is observed for *E. coli* and *B. subtilis*, with mannan exerting a significant inhibitory effect on binding. Conversely, in cases involving structural alterations in the lung tissue, such as pulmonary cavitary disease (PCD) and bronchiectasis, there appears to be a shift in macrophage activity, leading to their active engagement with normal bacteria, specifically *L. fermentum*, while a lesser effect of mannan is observed.

## 4. Conclusions

Macrophages’ phagocytic activity is considered here as an important parameter for categorizing Mph extracted from the BALF of patients with inflammatory conditions (PCD, asthma and pneumonia) in comparison with the reference CD206+ macrophages (M1) derived from model human THP-1 monocytes. This approach can be applied to characterize the Mph polarization state, subpopulation and phenotype. Here, we focus on macrophages isolated from BALF that interact with five distinct strains of *Escherichia coli* expressing red fluorescent protein (RFP), both in the presence and absence of competing *Lactobacilli*. CD206-positive Mph cells were isolated from BALF using a two-step approach involving the use of a polymeric ligand—a “trap for macrophages” followed by an “imaging” ligand for subsequent cellular imaging. The identification of Mph expressing the CD206 receptor was achieved through the application of fluorescence microscopy, complemented by FTIR microscopy. This approach allowed for the mapping and determination of the spatial distribution of receptors on the Mph surface.

A fluorometric analysis of the kinetics of the phagocytic response of Mph towards *E. coli* was conducted in the presence or absence of *Lactobacilli* as competing cells. The rate of *E. coli* adhesion to macrophages was determined by measuring RFP fluorescence. In the absence of *Lactobacillus*, *E. coli* was captured by macrophages at a rate corresponding to approximately four to six bacterial *E. coli* cells adhering to each Mph cell in hour. In the presence of *Lactobacilli* as a competitor, the binding capacity was reduced by two times. A clear correlation is observed between the minimum inhibitory concentration (MIC) of an antibiotic and the capacity of macrophages to attach to these microorganisms.

In contrast to the fluorescence method, which provides information on the *E coli* cells both bound and absorbed by Mph, CLSM was applied to determine the intracellular absorption. The average number of phagocytized bacteria per one Mph cell ranges from three to six (depending on the cell lines), indicating the relatively low phagocytic capacity of BALF-derived Mph. Apparently, in the presence of *Lactobacilli*, the phagocytic capacity of Mph against *E. coli* strains increases to up to 30 phagocytized bacteria per Mph, which implies the specific activation of Mph after 4–6 h of incubation in the presence of *Lactobacilli* cells.

The number of the relative phagocytic activity indexes obtained by different methods for BALF-derived Mph in comparison with model human CD206+ Mph in the M1 polarization state derived from THP-1 monocyte cultures used as a reference model can be considered as set of parameters to define the macrophage polarization profile from the BALF of patients.

Mannan significantly inhibited the phagocytosis of *E. coli* and *B. subtilis* in case of pneumonia, suggesting CD206 overexpression plays a role in acute inflammation. Conversely, *L. fermentum* binding was elevated in case of PCD, potentially reflecting altered macrophage responsiveness in chronic lung diseases.

Therefore, the analysis of Mph isolated from BALF with the use of a set of polymeric ligands—a “trap for Mph” followed by an “imaging” ligand—for subsequent cellular imaging in comparison with the reference CD206+ Mph presents a valuable analytic tool for profiling the Mph from the BALF of patients with inflammatory conditions. These insights may hold significant potential for future applications in diagnostics and lead to the adjustment of therapeutic strategies for a variety of inflammatory conditions, such as purulent bronchitis, asthma, PCD and pneumonia.

## Figures and Tables

**Table 1:** Bacterial strains of *Escherichia coli* expressing red fluorescent protein (RFP), including confocal laser scanning microscopy (CLSM) images, transmission electron microscopy (TEM) images and photographs of colonies grown on agar plates. Data on the minimum inhibitory concentration (MIC) of levofloxacin (LF) in relation to each strain. The medium used was LB broth (pH 7.2) with 0.1 mg/mL ampicillin added. The incubation temperature was 37 °C.

Strain Expressing RFP	RFP Confocal Fluorescence Image	TEM Image	Photos of Colonies Grown on Agar Plates	MIC(LF), mg/L
*E. coli* MH-1	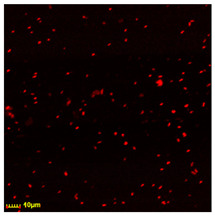	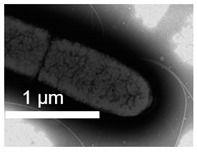	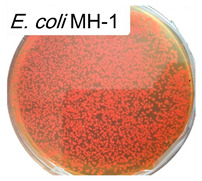	0.10 ± 0.02
*E. coli* JM109	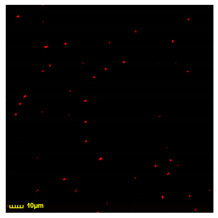	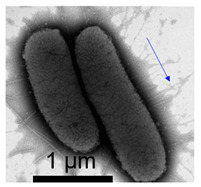	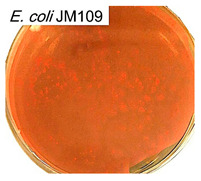	1.0 ± 0.1
*E. coli* BL21	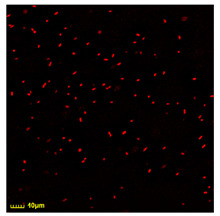	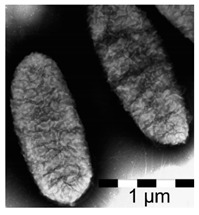	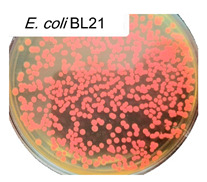	1.4 ± 0.2
*E. coli* W3110	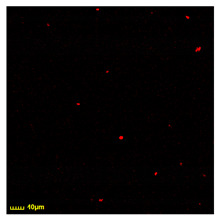	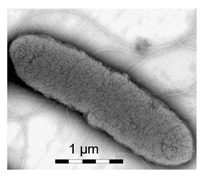	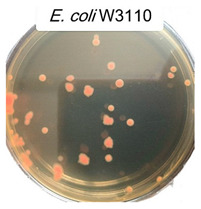	1.8 ± 0.2
*E. coli* ATCC25922	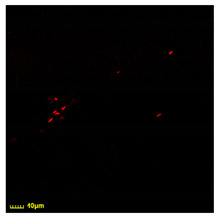	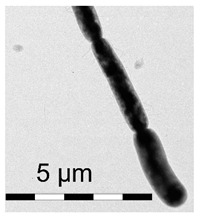	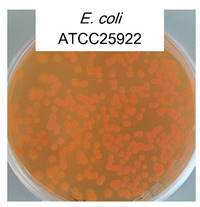	2.4 ± 0.3

**Table 2:** The physico-chemical properties of both the trapping ligand and the imaging ligand for CD206+ macrophages. PBS (pH 7.4, 0.01 M). T = 37 °C.

Ligand	Chemical Designation	Polymer	Molecular Weight of Ligand, kDa	Hydrodynamic Size *, nm	ζ-Potential **, mV	*K*_dis_ (ConA–Ligand), M ***	% of FITC-Labelled CD206+ Macrophages ****
**Trapping**	trimannoside-PEG (1:1 mol/mol)	PEG 5 kDa	6 ± 1	90 ± 10	+1.5 ± 0.5	(8 ± 1) × 10^−7^	73 ± 2
**Imaging**	HPCD-PEI-trimannoside-FITC (2:1:0.5:5 by mol)	PEI 2 kDa	9.7 ± 1.5	115 ± 20	+7 ± 1	(2.5 ± 0.5) × 10^−7^	80 ± 4

* by NTA. ** by DLS. *** ConA—is a model CD206 receptor. **** After 2 h of incubation with the ligands.

**Table 3:** Quantitative data on the interaction of *E. coli* bacteria with macrophages, including the total bacterial uptake over a 2 h incubation period and the initial phagocytic rate. The experimental conditions were similar to those described in the legend of Figure 3.

**Without *Lactobacilli* competitor**					
**Strain**	***E. coli* MH-1**	***E. coli* JM109**	***E. coli* BL21**	***E. coli* W3110**	***E. coli* ATCC25922**
*E. coli* adsorbed and absorbed by macrophages in 2 h, % (from added)	11 ± 1	14 ± 2	14 ± 1	19 ± 2	20 ± 2
The initial rate of *E. coli* binding, %/min	5.0 ± 0.5	3.7 ± 0.8	4.3 ± 0.6	4.5 ± 0.5	5.1 ± 0.7
***Lactobacilli* competitor**					
**Strain**	***E. coli* MH-1**	***E. coli* JM109**	***E. coli* BL21**	***E. coli* W3110**	***E. coli* ATCC25922**
*E. coli* adsorbed and absorbed by macrophages in 2 h, % (from added)	10 ± 1	11 ± 1	10 ± 1	11 ± 1	14 ± 2
The initial rate of *E. coli* binding, %/min	4.1 ± 0.3	4.7 ± 0.4	1.4 ± 0.1	1.5 ± 0.2	4.4 ± 0.8

## Data Availability

The data presented in this study are available in the main text and in Supplementary Materials.

## Supplementary Materials

The following supporting information can be downloaded at: https://www.mdpi.com/article/10.3390/polym17010065/s1, Figure S1: (a) The scheme of synthesis of the trimannoside-PEG (triMan–PEG) trapping ligand. (b) FTIR spectra of the initial substances and the target product. PBS (0.01 M, pH 7.4). T = 22 °C. Figure S2: (a) Scheme of synthesis; and (b) FTIR spectrum of FITC-labeled HPCD-PEI-triMan ligand for macrophage imaging. PBS (0.01 M, pH 7.4). T = 22 °C. Figure S3. Confocal laser scanning microscopy images of CD206+ macrophages derived from BALF after 4h-incubation with different *E. coli* cells in presence of *Lactobacilli* competitor. Macrophage were stained with FITC anti-CD206 ligand (HPCD-PEI1.8-triMan-FITC)—green channel (λ_ex,max_ = 488 nm; λ_emi_ = 505–555 nm). *E. coli* cells were stained RFP—red channel (λ_ex,max_ = 559 nm; λ_emi_ = 575–625 nm). *Lactobacilli* cells were stained methylene blue (MB)—cyan channel (λ_ex,max_ = 635 nm; λ_emi_ = 650–750 nm).
