# Supplementary material for: Optical Methods for Determining the Phagocytic Activity Profile of CD206-Positive Macrophages Extracted from Bronchoalveolar Lavage by Specific Mannosylated Polymeric Ligands
Source: Polymers (Basel). 2024 Dec 30;17(1):65. doi: 10.3390/polym17010065 (PMC11723180; doi:10.3390/polym17010065)

Article

Optical Methods for Determining the Phagocytic Activity profile of CD206-positive macrophages Extracted from Bronchoalveolar Lavage by Specific Mannosylated Polymeric Ligands

Igor D. Zlotnikov [1], Alexander A. Ezhov [2], Natalia I. Kolganova[3], Dmitry Yu. Ovsyannikov[3], Natalya G. Belogurova [1] and Elena V. Kudryashova [1,*]

[1] Faculty of Chemistry, Lomonosov Moscow State University, Leninskie Gory, 1/3, 119991 Moscow, Russia; zlotnikovid@my.msu.ru, nbelog@mail.ru (N.G.B.)

[2] Faculty of Physics, Lomonosov Moscow State University, Leninskie Gory, 1/2, 119991 Moscow, Russia; alexander-ezhov@yandex.ru (A.A.E.)

[3] Federal State Autonomous Educational Institution of Higher Education "Patrice Lumumba Peoples' Friendship University of Russia", 6, Miklukho-Maklaya str., Moscow, 117198

* Correspondence: helena_koudriachova@hotmail.com (E.V.K.)

Figure S1. (**a**) The scheme of synthesis of the trimannoside-PEG (triMan–PEG) trapping ligand. (**b**) FTIR spectra of the initial substances and the target product. PBS (0.01 M, pH 7.4). T = 22 °C.

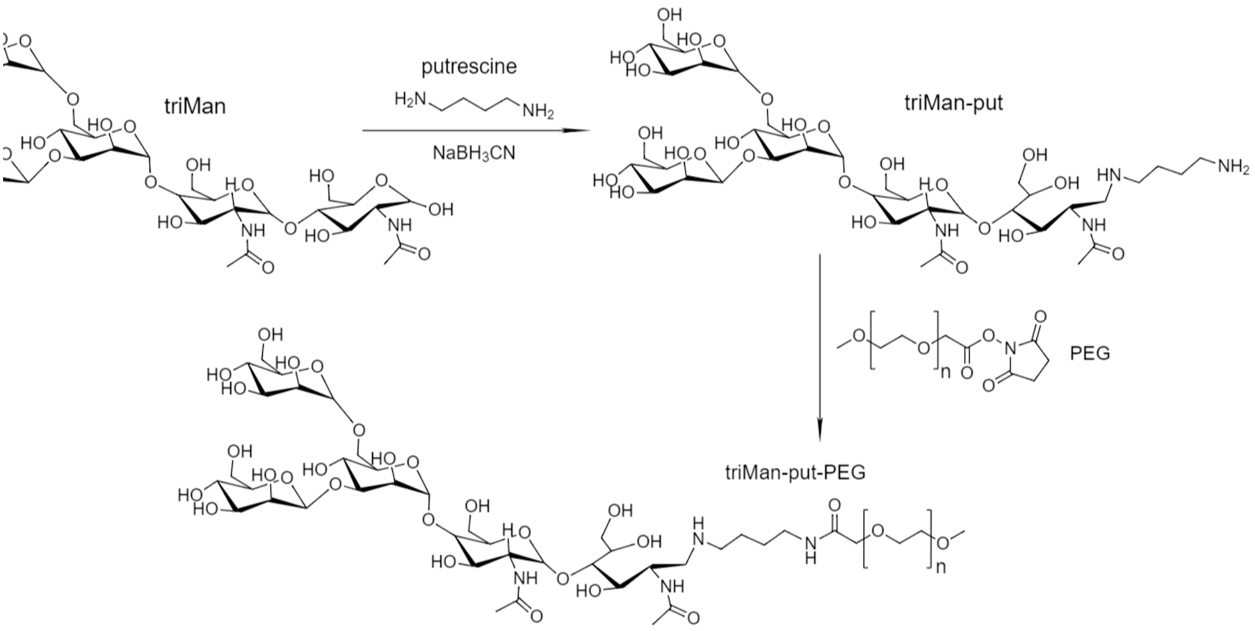

(**a**)

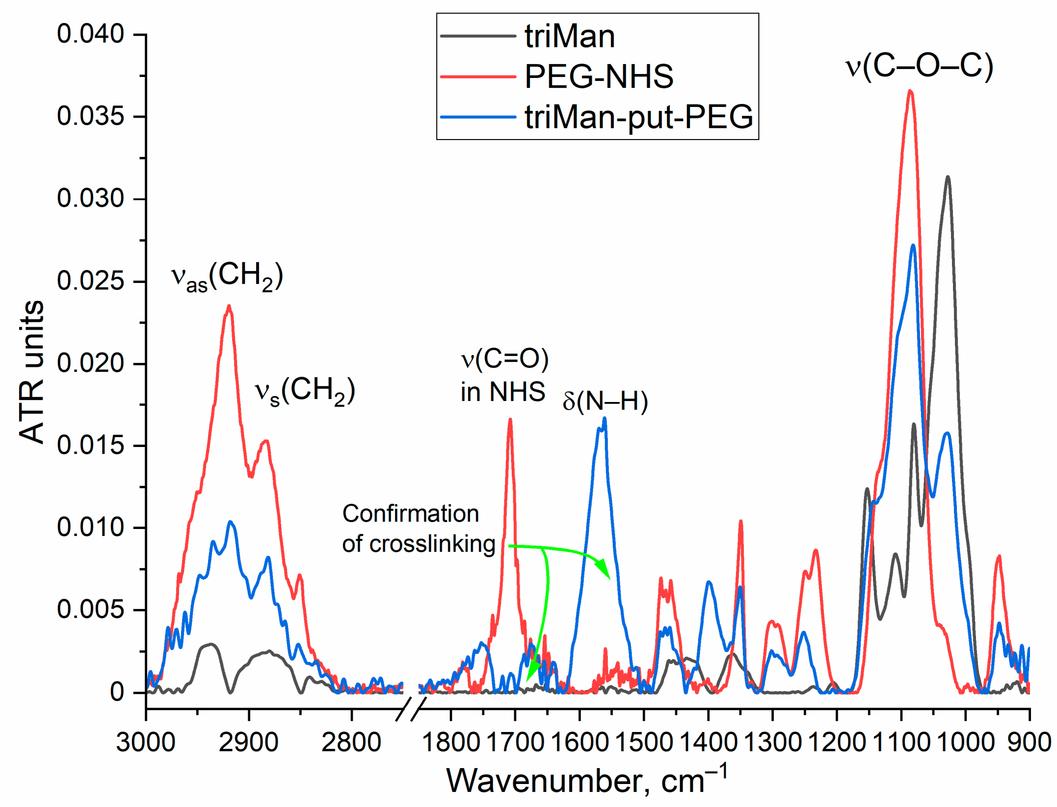

(**b**)

Figure S2. (a) Scheme of synthesis and (b) FTIR spectrum of FITC-labeled HPCD-PEI-triMan ligand for macrophage imaging. PBS (0.01 M, pH 7.4). T = 22 °C.

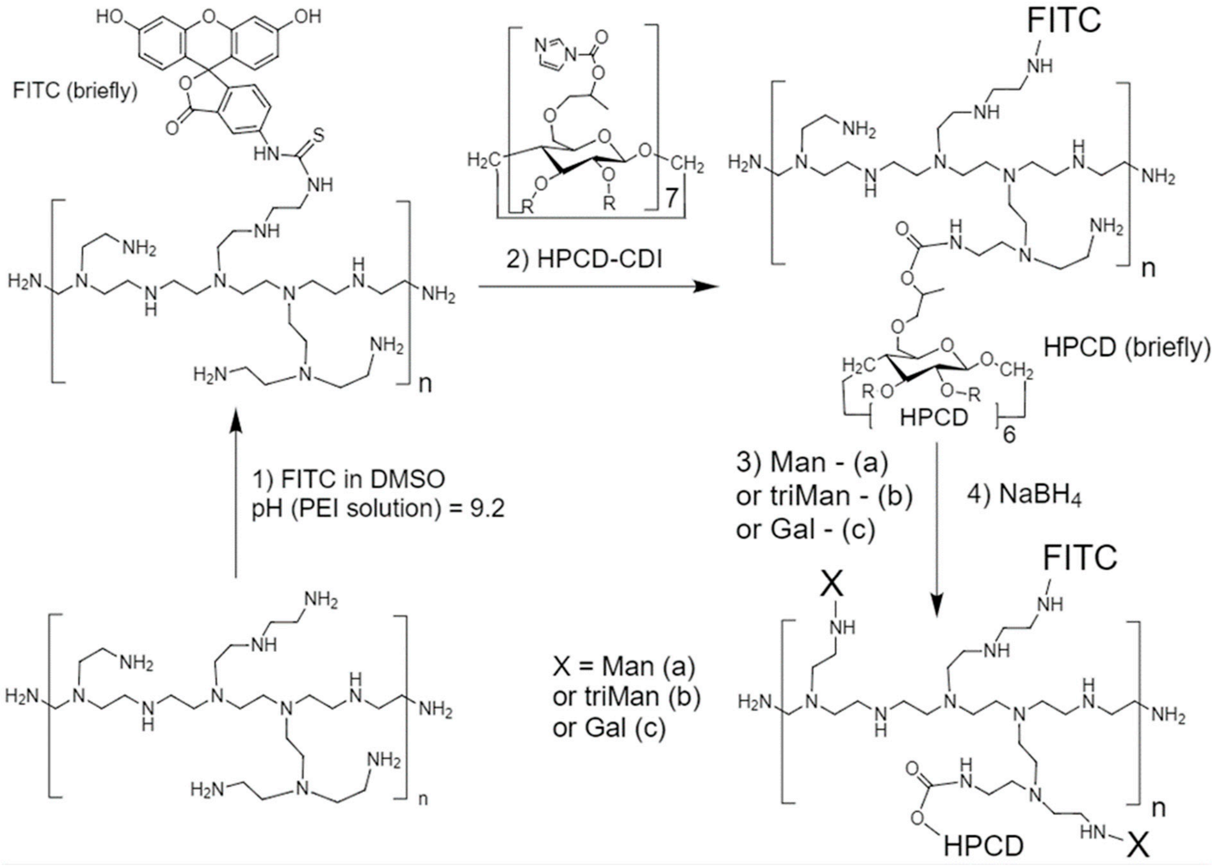

(a)

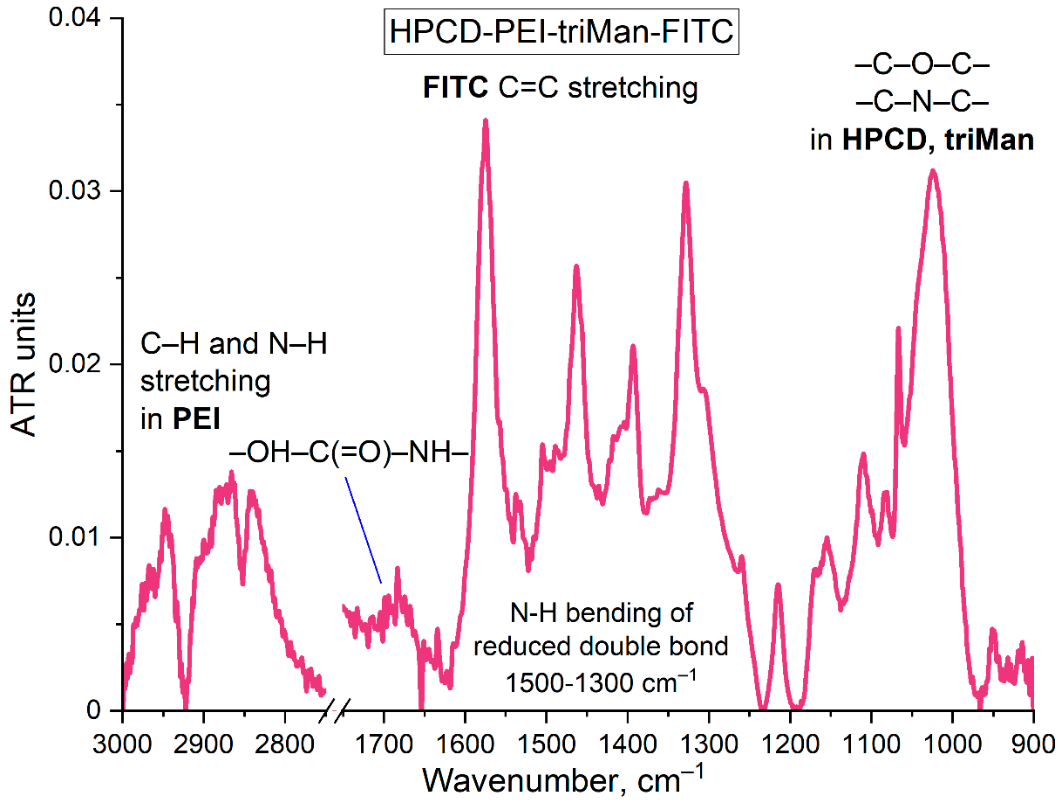

(b)

Figure S3. Confocal laser scanning microscopy images of CD206+ macrophages derived from BALF after 4h-incubation with different *E. coli* cells in presence of *Lactobacilli* competitor. Macrophage were stained with FITC anti-CD206 ligand (HPCD-PEI1.8-triMan-FITC) – green channel ($\lambda_{ex,max}$ = 488 nm; λ_{emi} = 505-555 nm). *E. coli* cells were stained RFP – red channel ($\lambda_{ex,max}$ = 559 nm; λ_{emi} = 575-625 nm).). *Lactobacilli* cells were stained methylene blue (MB) – cyan channel ($\lambda_{ex,max}$ = 635 nm; λ_{emi} = 650-750 nm).

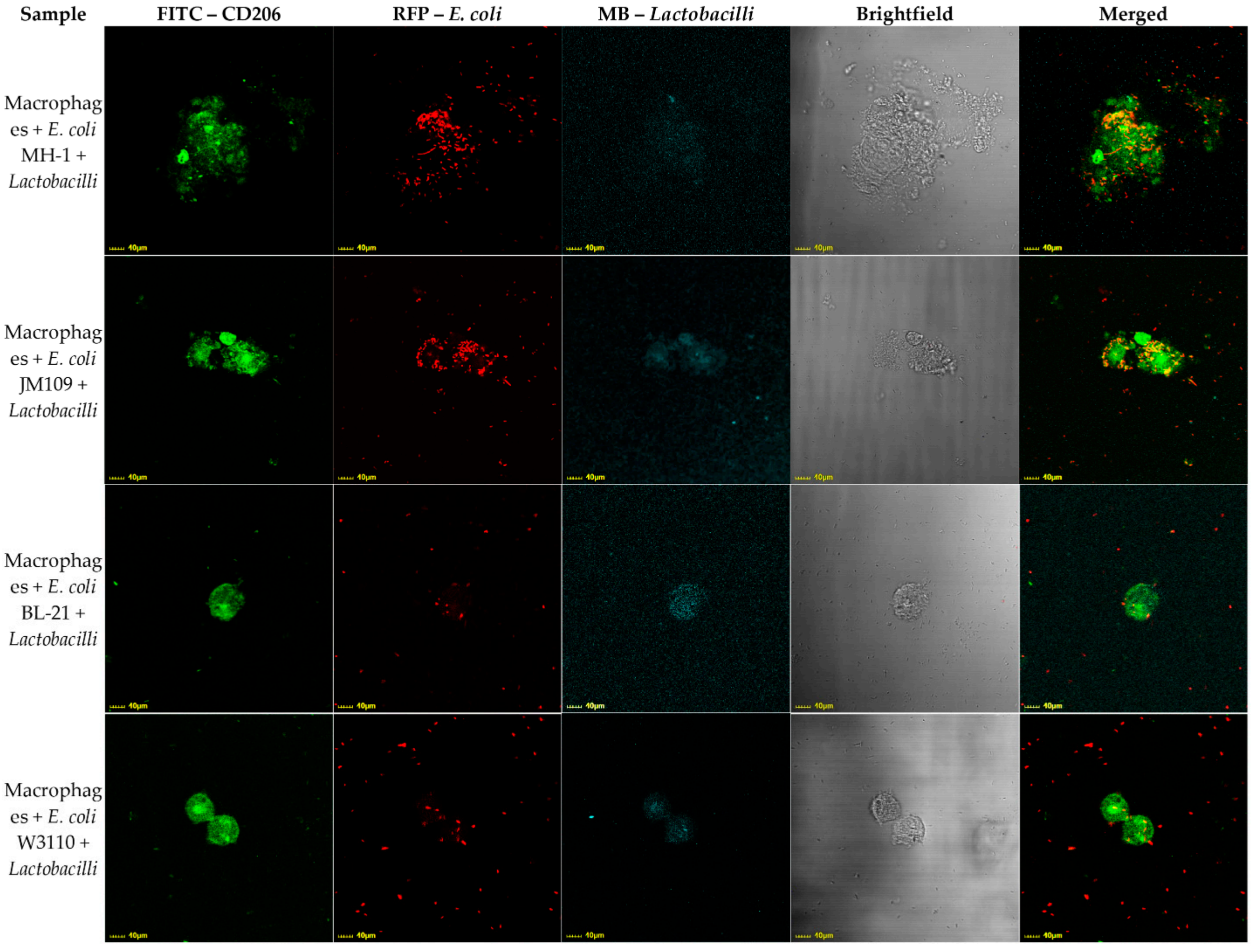

Supplement: Supplementary file 1 [file polymers-17-00065-s001.zip › polymers-3396966-supplementary/polymers-3396966-supplementary.pdf]
